# Evaluation of myeloid-related protein 126, cardiac troponin C and serum amyloid A as potential plasma biomarkers of health and disease in sea turtles

**DOI:** 10.1093/conphys/coaf061

**Published:** 2025-08-14

**Authors:** David P Marancik, Christopher C Chadwick, Paul Fields, Charles A Manire, Terry M Norton, Justin R Perrault, Carolyn Cray

**Affiliations:** Department of Pathobiology, School of Veterinary Medicine, St. George's University, True Blue, Grenada, West Indies; Life Diagnostics, Inc., 124 Turner Lane, West Chester, PA, 19380, USA; Windward Islands Research and Education Foundation, St. George, Grenada; Loggerhead Marinelife Center, 14200 U.S. Highway One, Juno Beach, FL, 33408, USA; Georgia Sea Turtle Center, Jekyll Island Authority, 214 Stable Road, Jekyll Island, GA, 31527, USA; Loggerhead Marinelife Center, 14200 U.S. Highway One, Juno Beach, FL, 33408, USA; Division of Comparative Pathology, Department of Pathology and Laboratory Medicine, University of Miami Miller School of Medicine, 1600 NW 10th Avenue, Miami, FL 33136, USA

**Keywords:** Biomarkers, cardiac troponin C, health, MRP-126, plasma, sea turtles, serum amyloid A

## Abstract

Sea turtle health assessments can be strengthened by developing conserved biomarkers that discriminate between healthy and diseased states. Serum amyloid A, myeloid-related protein 126 and cardiac troponin C (CTNC) were explored as potential biomarkers of sea turtle health. Plasma concentrations initially quantified using a targeted SPARCL™ assay significantly differed between moribund (*n* = 15) and recovered (*n* = 5) loggerhead turtles (*Caretta caretta*). There was a negative correlation between myeloid-related protein 126 and packed cell volume (*r* = −0.612, *P* = 0.005) and total solids (*r* = −0.497, *P* = 0.03) and between and Fulton’s body condition index (*r* = −0.684, *P* = 0.001). Serum amyloid A showed a relatively high interquartile range (IQR) in moribund turtles and no significant correlations with clinical parameters. Myeloid-related protein 126 and cardiac troponin C were further evaluated by an enzyme-linked immunosorbent assay in a larger dataset of loggerhead, Kemp’s ridley (*Lepidochelys kempii*) and green (*Chelonia mydas*) turtles. Plasma myeloid-related protein 126 was significantly lower in captive healthy (*n* = 7) and recovered (*n* = 23) turtles than in moribund (*n* = 25) and nesting green (*n* = 58) turtles. Green turtles with fibropapillomatosis (*n* = 10) were not significantly different from any group. Discriminating values between healthy/recovered and moribund turtles were 1.89 and 1.97 ng/ml by receiver operating characteristic and logistic regression analyses, respectively. Myeloid-related protein 126 decreased in successfully rehabilitated turtles (*n* = 18 turtles; *n* = 67 blood samples) and was negatively correlated with body condition score (*r* = −0.672, *P* < 0.001) and packed cell volume (*r* = −0.443, *P* = 0.009). Cardiac troponin C was significantly higher (*P* = 0.049) in moribund turtles (*n* = 16) compared to healthy/recovered turtles (*n* = 7) and in moribund samples (*n* = 11) compared to recovered samples (*n* = 11) in serially sampled turtles (*P* = 0.015), but was not predictive of health status. Myeloid-related protein 126 represents a strong biomarker candidate in sea turtles. Cardiac troponin C warrants further evaluation in a larger dataset and serum amyloid A requires examination of variables affecting pathophysiologic responses in sea turtles.

## Introduction

Sea turtle populations remain at risk with six of seven species listed as vulnerable, endangered or critically endangered (IUCN, 2024). An essential component of managing and conserving these populations is accurate evaluation of sea turtle health. This includes population-level assessments to monitor health trends over time (Page-Karjian *et al*., 2020; Molter *et al*., 2022; Soehnlein *et al*., 2022), detecting environmental impacts on health including climate change and ecotoxicology (Chaousis *et al*., 2023; Charles *et al*., 2023; Fernández-Sanz *et al*., 2023; Vanstreels *et al*., 2023) and diagnosing disease and managing treatment of debilitated and injured turtles (Jakšić *et al*., 2022; Omedes *et al*., 2023). Monitoring health indices can improve measures to assess risks and impacts associated with infectious, toxicologic and anthropogenic risks and inform decision-making regarding the conservation of threatened species (Kock, 1996; Kophamel *et al*., 2022).

A number of approaches are currently utilized to characterize sea turtle health in clinical and research settings. Complete blood counts (CBC) and plasma biochemistry have historically provided important physiologic data associated with health and disease (Herbst and Jacobson, 2003; Fernández-Sanz *et al*., 2023; Hayakijkosol *et al*., 2024; Kucinick *et al*., 2024). Blood gas analysis utilizing point-of-care instruments are valued to assess gas exchange and metabolic processes (Harms *et al*., 2003; Keller *et al*., 2012; McNally *et al*., 2020). Although CBC, biochemistry and blood gases provide useful data for assessing health, these methods were initially designed for mammalian species, and analytical techniques and parameters do not always translate to functional measurements of important pathophysiologic processes of sea turtles (Müller and Brunnberg, 2009). Additionally, reference intervals for healthy turtles can vary between species, life-stage classes, sex, diet and environment, which can limit data interpretation across different sea turtle aggregations (Deem *et al*., 2009; Anderson *et al*., 2011; Kucinick *et al*., 2024). Other avenues used to characterize sea turtle health include molecular markers of stress (Page-Karjian *et al*., 2020; Morão *et al*., 2022; Omedes *et al*., 2023; Rivas-Hernández *et al*., 2024) and cellular phagocytosis and oxidative burst assays to assess immune responses (Rossi *et al*., 2016; Perrault *et al*., 2021a, 2021b). These data provide quantified parameters to evaluate host responses but are challenging to run in clinical settings.

Advancements in biotechnology continue to introduce new dimensions for exploring disease responses in sea turtles. Expanded opportunities for more in-depth analysis of sea turtle health include elucidation of select sea turtle genomes (Bentley *et al*., 2023; Chang *et al*., 2023; Guo *et al*., 2023) and transcriptomes (Banerjee *et al*., 2021) and associated mass spectrometry driven ‘omics’ platforms. Proteomic (Marancik *et al*., 2021; Bianchi *et al*., 2022; Chaousis *et al*., 2023), lipidomic (Ahmadireskety *et al*., 2020; Clyde-Brockway *et al*., 2021), metabolomic (Niemuth *et al*., 2020; Melvin *et al*., 2022) and transcriptomic (Blackburn *et al*., 2021) studies have generated extensive datasets revealing biologic and physiologic systems of sea turtles during healthy and diseased states. These data also provide a platform to explore novel biomarkers focused on sea turtle pathophysiology. Specifically, selecting plasma proteins in omic-based platforms that show differential regulation between healthy and diseased states may reveal novel diagnostic indicators. The benefit of plasma proteins is that they can be sampled antemortem and via minimally invasive venipuncture in the field and in veterinary settings.

This study explored the utility of serum amyloid A (SAA), myeloid-related protein 126 (MRP-126) and cardiac troponin C (CTNC) as potential biomarkers of sea turtle health using targeted protein-specific methodologies. Serum amyloid A and MRP-126 were previously characterized in plasma of five moribund and five clinically recovered green turtles using a TMT-10 plex proteomics platform (Marancik *et al*., 2021). Serum amyloid A demonstrated increased plasma concentrations of up to 100-fold in moribund compared to healthy green turtles although differential regulation was not consistent between turtles within each clinical group (Marancik *et al*., 2021). Serum amyloid A is a highly conserved acute-phase protein of vertebrates (Sack, 2018) and has been characterized in nesting Kemp’s ridley (*L. kempii*) and loggerhead (*C. caretta*) sea turtles (Cray and Stacy, 2015) and associated with disease processes in soft-shelled turtles (*Trionyx sinensis*) (Zhou *et al*., 2011) and African tiger snakes (*Telescopus semiannulatus*) (Burns *et al*., 2017).

Proteomic analysis demonstrated MRP-126 concentrations to be 1.9 times higher in the plasma of green turtles presenting with buoyancy issues and 15.2 times higher in turtles presenting with trauma compared to samples when turtles were recovered (Loes *et al*., 2018). The MRP-126 gene found in reptiles and birds is an orthologue to mammalian S100-A12 belonging to the S100/calgranulin family of proteins (Loes *et al*., 2018) and S100-A12 is considered a pro-inflammatory modulator used as a biomarker of cellular damage, trauma and sepsis in mammals (Meijer *et al*., 2012; Khorramdelazad *et al*., 2015; Dubois *et al*., 2019; Zornow *et al*., 2023). MRP-126 has been associated with intestinal inflammation in chickens (Dal Pont *et al*., 2021) with demonstrated antimicrobial properties (Bozzi and Nolan, 2020). A sample size of three clinically abnormal Aldabra giant tortoises (*Aldabrachelys gigantea*) demonstrated increased concentrations of MRP-126 compared to a reference population of 27 individuals (Dianis *et al*., 2025). S100-A6 is upregulated in the skin of loggerheads experimentally exposed to the toxicant mono-(2-ethylhexyl) phthalate (Nash and Ryan, 2023) suggesting S100 family proteins have similar inflammatory properties between mammals, birds and sea turtles.

Cardiac troponin C is a regulatory protein involved in calcium-dependent contraction of cardiac and slow-twitch skeletal muscle (O'Connell *et al*., 2004; Li and Hwang, 2015) and is conserved between mammalian, bird, amphibian and fish species (Gillis *et al*., 2007). Increased plasma concentrations have been associated with myositis in mammals (Sleeper *et al*., 2001) and fish (Wiens *et al*., 2023) and although not currently described in sea turtles, similar trends may be present associated with non-specific cardiac and skeletal muscle damage and protein leakage into the circulatory system.

Elucidating relative plasma concentrations of SAA, MRP-126 and CTNC between healthy and disease states and exploring associations with known clinical and biologic variables provide a clinical context for exploring these proteins as potential diagnostic biomarkers of sea turtles. It also establishes a new quantitative assay to measure novel protein analytes in sea turtles that may further benefit research and health monitoring of these ecologically valuable animals.

## Materials and Methods

This study was approved by the St. George’s University Institutional Animal Care and Use Committee (IACUC-24008-R). Applicable permits were obtained from the Florida Fish and Wildlife Commission (FWC) (MTP-211, MTP-125 and MTP-139) and National Marine Fisheries Service (NMFS) (16598).

### First clinical study, spatial proximity analyte reagent capture luminescence assay

#### Sample population and clinical assessment

Frozen plasma and retrospective clinical data were examined from one juvenile and 19 subadult, free-ranging loggerheads that presented to the Loggerhead Marinelife Center (LMC; Juno Beach, FL, USA) for treatment and rehabilitation between 2016 and 2019 (FWC permit MTP-211). Plasma and clinical data were obtained from 15 moribund turtles upon initial presentation to LMC with hook ingestion, entanglement or Chronic Debilitation Syndrome (CDS) defined as emaciation, lethargy and heavy epibiota with largely unknown specific causes (Stacy *et al*., 2018) (Table 1). Five clinically recovered turtles comprised a second dataset. Plasma samples and clinical data were collected prior to release when turtles were interpreted to be clinically healthy based on resolution of clinical signs and bloodwork abnormalities and observations of mobility and appetite.

**Table 1:** Summary of characteristics of sample population for each dataset

**Study dataset**	**Status sea turtle species**	**Class size/sex**	**Presenting etiologies**
First Study, SPARCL™ Assay			
	Recovered Loggerhead, *n* = 5 Moribund Loggerhead, *n* = 15	Juvenile Juvenile	Chronic debilitation syndrome, *n* = 12 Entanglement, *n* = 1 Hook ingestion, *n* = 2
Second Study, ELISA			
Single sample	Healthy Loggerhead, *n* = 5 Green turtle *n* = 1 Recovered Loggerhead, *n* = 4 Green turtle, *n* = 2 *Moribund* Loggerhead, *n* = 3 Green turtle, *n* = 2 Kemp’s ridley, *n* = 1	Juvenile Juvenile Juvenile, *n* = 6 Subadult, *n* = 1 Juvenile Juvenile Juvenile Juvenile	Pneumonia, *n* = 2 Chronic debilitation syndrome, *n* = 2 Buoyancy disorder, *n* = 2 Trauma, *n* = 3 Mycobacteria, *n* = 1
Serial sample	Healthy Loggerhead, *n* = 1 Treated Loggerhead, *n* = 3 Green turtle, *n* = 7 Kemp ridley, *n* = 9	Juvenile Juvenile, *n* = 1 Adult, female, *n* = 2 Juvenile Juvenile	Anaemia, *n* = 1 Trauma, *n* = 4 Fibropapillomatosis/pneumonia, *n* = 1 Cold stunned/pneumonia/trauma, *n* = 6 Cold stunned/pneumonia, *n* = 4 Cold stunned/trauma, *n* = 2 Cold stunned/fibropapillomatosis/trauma, *n* = 1
Nesting	Green turtle, *n* = 58	Female	Apparently healthy, *n* = 58
Fibropapillomatosis	Green turtle, *n* = 10	Juvenile	Fibropapillomatosis, *n* = 10

The sex of juvenile and subadult turtles is undetermined due to lack of morphological sex characteristics.

Each turtle was physically examined at the time of venipuncture and morphometric measurements were collected including standard straight carapace length (SCL), straight carapace width (SCW) and mass (Supplemental File 1). Life-stage classes (e.g. juvenile, subadult) were estimated based on SCL (Southeast Fisheries Science Center (U.S.), Turtle Expert Working Group, 2009). Fulton’s body condition index (BCI) was calculated using the formula: body mass/(SCL)^3^ (Bjorndal *et al*., 2000). The five turtles sampled prior to release were maintained during rehabilitation in outdoor 1200- to 3600-l fibreglass tanks on a flow-through system utilizing natural seawater at ambient ocean temperature except that in winter the incoming water was heated when needed to maintain the water temperature >22°C. In addition to a daily diet of shrimp or squid and capelin, turtles were supplemented with Mazuri® Sea Turtle Supplements and calcium (Risacal-D, Rising Pharmaceuticals Inc., Allendale, NJ, USA).

Blood samples (<1% body mass) were collected at presentation or prior to release from the external jugular vein using a 22-gauge, 1-in needle and 3-cm^3^ syringe and transferred to lithium heparin tubes. Total white blood cell counts were estimated using Avian Leukopet White Blood Cell Kit (Palmeto Bay, FL, USA) and differential leukocyte counts were performed manually from blood smears under light microscopy. Packed cell volume (PCV) was determined in microhaematocrit tubes after centrifugation for 5 min at 1600 *g* (4000 rpm) using a Hettich EBA270 centrifuge (Tuttlingen, Germany). Total solids (TS) were quantified using a refractometer (BK-PR series, Biobase, China). Remaining blood was centrifuged at 4200 *g* (5000 rpm) for 8 min using an LW Scientific C5 Centrifuge (Lawrenceville, Georgia, USA). Plasma biochemistry was performed on a portion of the plasma sample using an IDEXX VetTest Chemistry Analyser (Westbrook, ME, USA) (Supplemental File 1). Clinical evaluations were based on the medical needs of each turtle and thus diagnostic data collected varied between some turtles and are listed in Supplemental File 1. Remaining plasma was stored at −80°C.

### Second clinical study, ELISA

#### Sample population and clinical assessment

Based on results from the First Clinical Study, MRP-126 and CTNC were prioritized for further evaluation using archived plasma and retrospective clinical datasets from loggerheads, Kemp’s ridleys and green turtles (*C. mydas*) (Table 1). MRP-126 data are listed in Supplemental File 2 consisting of four datasets. A subset of turtles within the Single and Serial Sampled Datasets were quantified for CTNC concentrations based on enzyme-linked immunosorbent assay (ELISA) reagent availability and data are listed in Supplemental File 2.

(1) ‘Single Sample Dataset’: A single blood sample was collected from captive healthy resident turtles (*n* = 6), moribund turtles sampled at presentation (*n* = 6) or recovered turtles sampled prior to release (*n* = 6) at the Georgia Sea Turtle Center (GSTC; Jekyll Island, GA, USA). MRP-126 concentrations were quantified in all samples and 11 turtles were evaluated for CTNC.
(2) ‘Serial Sample Dataset’: Serial blood samples were collected from individual turtles at presentation, throughout treatment at the GSTC and when clinically interpreted to be recovered (*n* = 19 turtles; *n* = 70 blood samples). This dataset also included four samples collected from one healthy loggerhead that was a permanent resident of the GSTC. Forty of these samples were evaluated for CTNC concentrations based on ELISA reagent availability.
(3) ‘Nesting Sea Turtle Dataset’: A single blood sample was collected from nesting green turtles during ovipositioning on Juno Beach (*n* = 58) (FWC permits MTP-139 and MTP-205).
(4) ‘Fibropapillomatosis Dataset’: A single blood sample was collected from green turtles visually confirmed with fibropapillomatosis (FP) that were caught during in-water health assessments in Big Bend, FL, USA, in 2020 (*n* = 10) (FWC permits MTP-125 and MTP-139 and NMFS permit 16598).

Cardiac troponin C was examined in: ‘Single Sample Dataset’: captive healthy resident turtles (*n* = 6), moribund turtles sampled at presentation (*n* = 4) and recovered turtles sampled prior to release (*n* = 1), and in the ‘Serial Sample Dataset’ (*n* = 14 turtles; *n* = 40 blood samples). The data are listed in Supplemental File 2a and b.

Turtles evaluated in the Single and Serial Sample Datasets presented to GSTC between 2021 and 2022. Rehabilitation of turtles was performed in 2.43 × 2.43 m or 3 × 3 m circular custom-made fibreglass tanks in 23–25°C filtered salt water. Filtration systems consisted of a protein skimmer, biological filter, bead filter and ozone for disinfection. Kemp’s ridleys and loggerheads were fed fish, squid and crabs, and green turtles received a primarily herbivorous diet of various greens and a custom gelatin-based diet consisting of vegetables, seafood and vitamins (Beth Firchau, Audubon Aquarium, unpublished data) with occasional seafood supplementation. All turtles received a multivitamin (Vita-Zu Sea Turtle Supplement, 0.5 g, Mazuri Exotic Animal Nutrition, St. Louis, MO, USA) and calcium supplement (calcium carbonate, 10-g tabs, Rugby, Livonia, MI, USA).

Morphometric measurements were recorded and a subjective body condition score (BCS) was assigned using a 1–5 scale. Venipuncture, PCV, TS and centrifugation to isolate plasma were performed as described above. Total leukocyte estimates were performed using a haemocytometer and Natt–Herricks stain (Divers, 2019) and differential counts were enumerated on blood smear cytology by ZooQuatic Laboratory, L.L.C (Baltimore, MD, USA). Biochemistry profiles were performed on plasma using standard dry-slide determinations with an Ortho 5600 Chemistry Analyser (Department of Pathology & Laboratory Medicine, University of Miami, Miami, FL, USA) using Vitros Performance Verifiers I and II (Ortho Diagnostics, Rochester, NY, USA) as quality control standards. Protein fractions were evaluated by plasma electrophoresis for turtles included in the Serial Sample Dataset (Zaias and Cray, 2002). Blood gas parameters were evaluated using an i-STAT Portable Clinical Analyser (Heska Corporation, Fort Collins, CO, USA) with CG8+ cartridges. The i-STAT device analysed the blood at 37°C and then pH, pCO_2_ and pO_2_ were corrected for body temperature (McNally *et al*., 2020). All examined diagnostic variables are listed in Supplemental File 2a and b.

Green turtles evaluated in the Nesting Sea Turtle Dataset were sampled once when encountered during ovipositioning on Juno Beach from June to August 2017 (Supplemental File 2c). Data were originally part of previously published health surveillance of nesting green turtles (Page-Karjian *et al*., 2020). Venipuncture, PCV, TS and plasma biochemistry were performed as outlined in the First Clinical Study and gel electrophoresis was performed as described above. Complete blood counts were estimated using blood smears. Presence of reactive oxygen species (ROS) and reactive nitrogen species (RNS) in plasma samples was evaluated using an OxiSelect™ *In Vitro* ROS/RNS Assay Kit (Green Fluorescence, Cell Biolabs, Inc., San Diego, CA, USA), while superoxide dismutase (SOD) activity was measured using commercially available assay kit (Cayman Chemical Co, Ann Arbor, MI, USA) following manufacturer’s instructions. Nest inventories were performed 3 days after the mass emergence event and the total number of eggs were counted and hatching and emergence success percentages were calculated.

Green turtles evaluated within the Fibropapillomatosis Dataset were captured and sampled from 27 to 30 August 2020 during in-water health assessments in Florida’s Big Bend (Perrault *et al*., 2021b) (Supplemental File 2d). Turtles were brought on board the research vessel and given an external physical examination (Page-Karjian *et al*., 2020) including morphometric measurements, Fulton’s BCI, BCS, enumeration of total tumour number and a Balazs–Work tumour score (Work and Balazs, 1999). Packed cell volume, TS, haematology and biochemistry were processed as described above and plasma was stored frozen at −80°C until analysis.

### Biomarker quantification

Plasma concentrations of SAA, MRP-126 and CTNC were determined using single-tube spatial proximity analyte reagent capture luminescence (SPARCL™) assays (Akhavan-Tafti *et al*., 2013) in the First Clinical Study or 96-well ELISAs in the Second Clinical Study developed by Life Diagnostics, Inc. (West Chester, PA, USA) and per manufacturer procedures. The SAA SPARCL ™ assay was originally developed for measurement of chicken-specific SAA using two peptide-specific antibodies targeting a region that shares 84% identity between chicken and green turtle SAA. The MRP-126 SPARCL™ and ELISA used rabbit polyclonal antibodies generated against recombinant green turtle MRP-126 using recombinant MRP-126 as a standard. The CTNC SPARCL™ and ELISA assays used antibodies against human CTNC with native human CTNC used as a standard. Human and green turtle CTNC share 96% identity. Dilutional linearity was demonstrated for all assays using turtle plasma with elevated concentrations of the respective proteins. Samples were analysed in duplicate. ELISA absorbance was read at 405 nm using a FLUOstar Omega reader (BMG LABTECH, Cary, NC, USA).

### Statistical analysis

The distribution of each dataset was evaluated using the Shapiro–Wilk test. Normal data distributions were observed for CTNC and MRP-126 but not SAA in the First Clinical Study and biomarker concentrations were compared between moribund and recovered turtles using a non-parametric Kruskal–Wallis test. A Spearman’s correlation compared relative concentrations of SAA, MRP-126 and CTNC in each turtle.

Within the Second Clinical Study, normal data distribution was observed for MRP-126 in datasets with healthy, recovered and moribund turtles but not nesting or FP green turtles and MRP-126 was compared between groups using a Kruskal–Wallis test. Samples comprising healthy, recovered and moribund turtles were compiled from the Single and Serially Sampled Groups. Within the Serially Sampled Group, the first sample was utilized as the moribund sample and the last sample as the recovered sample as multiple moribund and recovered blood samples were collected for several turtles (Supplemental File2). Additionally, one serially sampled green turtle (C22005) was euthanized due to poor response to treatment and thus no recovered blood sample was utilized in the study. Cardiac troponin C concentrations in healthy and recovered and moribund turtles were not normally distributed and data were compared between groups with a Mann–Whitney test. The first (moribund) and last (recovered) samples within the Serially Sampled Dataset were compared for both MRP-126 and CTNC with a Wilcoxon test. MRP-126 was further evaluated using healthy, recovered and moribund samples from the Single and Serially Sampled Datasets to determine the 95% confidence interval. MPR-126 and CTNC were examined by logistic regression to estimate the 50% probability of a turtle being considered healthy or moribund and receiver operating characteristic (ROC) curve was used to determine the accuracy, sensitivity and specificity to discriminate between healthy and moribund turtles. Both MRP-126 and CTNC were log-transformed prior to statistical analyses.

MRP-126 and CTNC concentrations were examined for associations with clinical parameters within the First and Second Clinical Studies (Supplemental Tables 1a and 2a) using Spearman’s correlation and simple linear regression using all available samples. Statistics were performed with GraphPad Prism 10 software (Boston, MA, USA) and MedCalc® Statistical Software version 23.0.9 (Ostend, Belgium). Differences were considered statistically significant when *P* < 0.050.

## Results

### First clinical study, SPARCL™ assay

**Figure 1:**
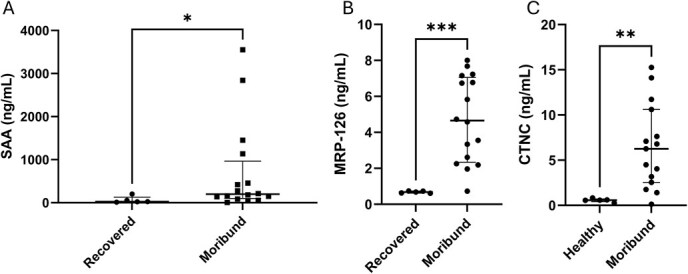
Median and IQR of plasma concentrations of (A) serum amyloid A (SAA), (B) MRP-126 and (C) CTNC in recovered and moribund loggerhead sea turtles (*C. caretta*) quantified by SPARCL™ assay. Asterisks indicate significant differences (*P = 0.026; ***P < 0.001; **P = 0.003).

Plasma concentrations of SAA, MRP-126 and CTNC were examined in 15 moribund and five recovered loggerheads. Plasma concentration of SAA, MRP-126 and CTNC were significantly higher in moribund turtles than recovered turtles (*P* = 0.026, *P* < 0.001 and *P* = 0.003, respectively) (Fig. 1). Pearson’s correlation showed a moderate positive correlation between SAA and MRP-126 concentrations in each turtle (r = 0.453, *P* = 0.045) and no correlations were present with CTNC.

**Figure 2:**
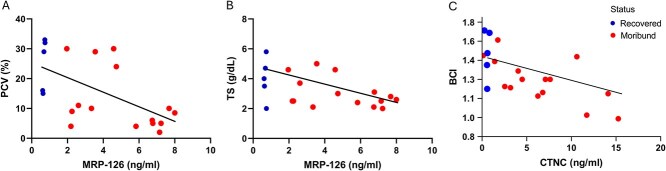
Simple linear regression of plasma concentrations of MRP-126 quantified by SPARCL™ assay and (A) PCV, (B) TS and CTNC and (C) BCI in recovered and moribund loggerhead sea turtles (*C. caretta*). Corresponding *P*-values: (A) *P* = 0.006, (B) *P* = 0.046 (C) *P* = 0.001.

Serum amyloid A showed no significant correlations with aetiology, morphometrics and clinical parameters collected from diagnostic bloodwork. MRP-126 had strong negative correlation with PCV (*r* = −0.612, *P* = 0.006) and a moderate negative correlation with TS (*r* = −0.497, *P* = 0.046). Subsequent linear regression analysis showed significant association with PCV (*P* = 0.006) and TS (*P* = 0.046) (Fig. 2a, b, respectively). Cardiac troponin C and BCI demonstrated a strong negative correlation (*r* = −0.684, *P* = 0.001) and a significant association by linear regression (*P* = 0.001) (Fig. 2c).

### Second clinical study, ELISA

**Figure 3:**
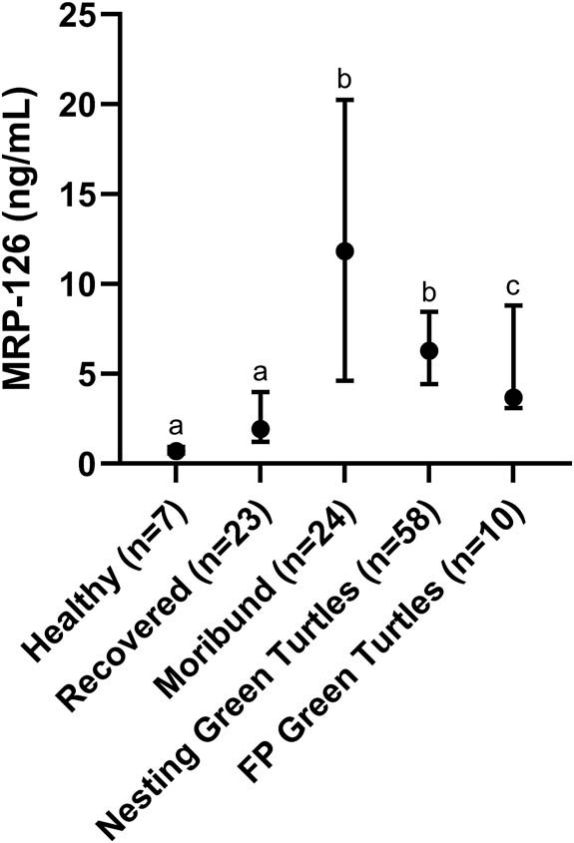
Median and IQR of MRP-126 concentrations in green (*C. mydas*), loggerhead (*C. caretta*) and Kemp’s ridley (*L. kempii*) turtles quantified by ELISA comprising captive healthy (0.71 ng/ml, 0.57–0.93), clinically recovered (1.92 ng/ml, 1.21–3.98) and moribund (11.83 ng/ml, 4.61–20.24) groups and green turtles that were nesting (6.29 ng/ml, 4.42–8.45) and wild-caught with FP (3.68 ng/ml, 3.10–8.80). Statistical groups are noted by letter. Significance was set to *P* < 0.050.

MRP-126 and CTNC were evaluated within the four different datasets composed of multiple species of sea turtles and clinical conditions. Median MRP-126 concentrations and IQRs for each dataset are presented in Fig. 3. When captive healthy and recovered turtles from the Single Sample and Serial Sample Datasets were evaluated collectively, plasma MRP-126 concentrations were not significantly different between groups (*P* > 0.999). Concentrations in moribund turtles were significantly higher in comparison to healthy (*P* = 0.001) and recovered turtles (*P* < 0.001). Concentrations in nesting green turtles were significantly higher than healthy and recovered turtles (*P* < 0.001 for both) but not moribund turtles (*P* = 0.762). There was no significant difference between concentrations in FP green turtles and healthy (*P* = 0.071), recovered (*P* = 0.777), moribund (*P* = 0.096) or nesting turtles (*P* > 0.999).

**Figure 4:**
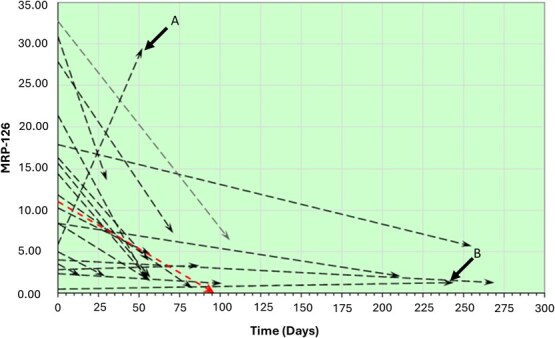
MRP-126 concentrations quantified by ELISA showed a mean downward trend (red arrow) for serially sampled turtles undergoing successful treatment and recovery. The exceptions were (A) one green turtle (*C. mydas*) (ID: C22005) that was euthanized after not responding to treatment and (B) one loggerhead sea turtle (*C. caretta*) (ID: C21298) that was a healthy resident turtle.

Non-parametric reference intervals indicated with 95% confidence that healthy/recovered turtles present with MRP-126 concentrations <5.0 ng/ml while moribund turtles have a concentration >2.1 ng/ml. The health status was unclear for turtles with concentrations between 2.1 ng/ml and 5.0 ng/ml. ROC analysis provided a threshold concentration of 1.89 ng/ml for MRP-126 as a discriminating value for heathy/recovered versus moribund turtles (*P* < 0.001) with an AUC of 0.953 (highly predictive), sensitivity of 100% and specificity of 82.4%. Threshold concentrations examined by logistic regression demonstrated the 50% probability of a turtle being considered moribund was 1.97 ng/ml. Using this model, the first sample from the Serial Sample Dataset correctly predicted 17/19 treated turtles as moribund with 14/17 having a >90% probability of being moribund. In the FP Dataset, 8/10 green turtles from the FP Dataset were categorized as moribund along with 57/60 green turtles from the Nesting Dataset. MRP-126 concentrations were significantly different between the first and last sample in serially sampled turtles (*P* = 0.001) and demonstrated a mean downward trend towards recovery during treatment. The exceptions within this dataset were one green turtle (C22005) that did not respond to treatment and was subsequently euthanized due to poor prognosis and one loggerhead (C21298) that was a healthy resident turtle (Fig. 4).

**Figure 5:**
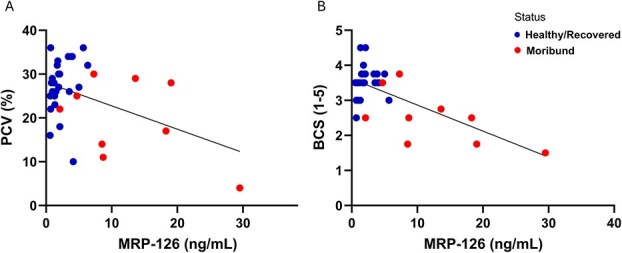
Simple linear regression of plasma concentrations of MRP-126 quantified by ELISA and (A) PCV and (B) subjective BCS in healthy/recovered and moribund sea turtles. Corresponding *P*-values: (A) *P* = 0.009, (B) *P* = 0.001.

The association of MRP-126 with available clinical parameters was examined in healthy and moribund turtles by combining the Single Sample Dataset and the last sample from turtles from the Serial Sample Dataset. No significant associations were observed between MRP-126 concentrations and disease aetiology or turtle species or size. There was a moderate negative correlation with PCV (*n* = 38, *r* = −0.443, *P* = 0.009) with significant association by linear regression (*P* = 0.009) (Fig. 5a, Supplemental File 2e). There was a strong negative correlation of MRP-126 with BCS (*n* = 38, *r* = −0.672, *P* < 0.001) and significant association by linear regression (*P* = 0.001) (Fig. 5b, Supplemental File 2e). Nesting green turtles and FP turtles demonstrated no significant correlations or associations between MRP-126 and morphometric or clinical parameters.

When concentrations of CTNC for healthy (median = 0.43 ng/ml, IQR = 0.10–1.45) and recovered (median = 0.10 ng/ml, IQR = 0.10–0.71) turtles were combined, they were significantly lower (*P* = 0.049) than moribund turtles (median = 0.68 ng/ml, IQR = 0.10–2.29). There was a significant difference (*P* = 0.015) between the first (moribund) and last (recovered) samples from turtles within the Serially Sampled Datasets; however, statistical analysis was largely influenced by two moribund samples of 110.98 and 59.11 ng/ml. The ROC curve model provided a discriminating value between healthy/recovered and moribund turtles of 0.09 ng/ml (*P* = 0.016) with an AUC value of 0.759, sensitivity of 81.3% and specificity of 71.4%. Similarly, a logistic regression model of the serially sampled turtles was shown to be significant (*P* = 0.025) but CTNC was not significant as a predictor of status (*P* = 0.080).

When the associations of CTNC with species, morphometrics and clinical parameters were examined within the combined Single Sample Dataset and the last (recovered) sample from turtles within the Serial Sample Dataset, there was a very strong negative correlation between CTNC and beta-globulins (*r* = −0.833, *P* = 0.022) and a significant association by linear regression (*P* = 0.022) (Supplemental File 2e). However, the sample size was relatively low (*n* = 7) and was significantly influenced by a single sample that when removed rendered the correlation (*P* = 0.614) and linear regression (*P* = 0.299) statistically insignificant. No significant associations were observed between CTNC concentrations and disease aetiology or turtle species or size.

## Discussion

The goal of this study was to explore the clinical utility of SAA, MRP-126 and CTNC as plasma biomarkers of health and disease in sea turtles. This study builds upon knowledge gained from previous proteomics results in green turtles (Marancik *et al*., 2021) by utilizing more targeted methodologies to quantify plasma protein concentrations in multiple sea turtle species at different life-stage classes and under various clinical conditions. Overall, MRP-126 demonstrated the most promise as a biomarker candidate as plasma concentrations discriminated between healthy/recovered and moribund turtles, showed decreasing trends in treated turtles that recovered, and had associations with clinical parameters such as BCI and PCV. An expanded clinical assessment of MRP-126 and further evaluation of CTNC and SAA are warranted to explore their utility as health biomarkers and to examine pathophysiologic responses in sea turtles.

Protein concentrations did not appear to be overtly associated with turtle species or size although statistically significant sample numbers representing life-stage classes of each species are needed to develop reference intervals. Traditional biomarkers quantified by plasma biochemistry and protein electrophoresis are well documented to vary between sea turtle species and secondary to water quality, life-stage class, diet and reproductive status (Osborne *et al*., 2010; Kucinick *et al*., 2024), which could also affect MRP-126, CTNC and SAA concentrations. There was strong evidence that MRP-126 concentrations are elevated in reproductively active female turtles and were statistically similar to moribund turtles compared to healthy/recovered turtles. It is unclear whether this elevation is due to vitellogenesis or nesting behaviour (e.g. reduced feeding, migration). Expression of MRP-126 and S-100 family proteins are described in avian (Qin *et al*., 2021) and human ovary (Liu *et al*., 2012; Jurewicz and Filipek, 2022), respectively, with elevated levels observed during ovulation and similar reproductive-induced inflammatory responses may be occurring in sea turtles. No correlations were evident between plasma MRP-126 concentrations and egg production or hatching or emergence success, and more in-depth studies are needed to examine the physiologic role of MRP-126 in reproductively active sea turtles. Subsequent to this finding, the caveat must be included that reproductive status may complicate diagnostic interpretation of MRP-126 in moribund turtles.

MRP-126 concentrations were highly predictive of morbidity when examined by ROC curve and logistic regression with consistent discriminating values of 1.89 and 1.97 ng/ml, respectively. Although species-specific considerations need to be taken into account, median MRP-126 concentrations in healthy/recovered turtles were consistent with a median value of 0.86 ng/ml described in a population of 27 healthy Aldabra giant tortoises, and two of three of these tortoises presenting with inflammatory or infectious conditions would have been categorized as moribund using the sea turtle ROC and logistic regression discriminating values (Dianis *et al*., 2025). More precise reference intervals would be beneficial in healthy turtles but the high predictivity of MRP-126 indicates its utility as a clinical biomarker in turtles. Additionally, there was consistently a progressive decrease of MRP-126 in serially sampled turtles that recovered and increased concentrations in one green turtle (C22005) that did not respond to treatment. This suggests that monitoring MRP-126 levels during treatment may be useful in monitoring patient progression along with identifying morbidity at presentation.

MRP-126 concentrations were not statistically associated with specific disease conditions (e.g. pneumonia, CDS, cold-stunning, trauma) suggesting it would be more applicable as a broad diagnostic marker rather than for aetiologic diagnosis. This is consistent with elevated concentrations of MRP-126 in avian species secondary to natural and experimental immune stimulation with bacteria (Meitern *et al*., 2014; Rychlik *et al*., 2014), viruses (Liu *et al*., 2019), parasites (He *et al*., 2019), heat stress (Park *et al*., 2022) and toxicoses (Dal Pont *et al*., 2021; Sun *et al*., 2022). Similarly, increased S100 in mammals is associated with a number of pathogenic processes including immune cell infiltration, neoplasia, traumatic injuries and neurologic and cardiac diseases (Sedaghat and Notopoulos, 2008). MRP-126 expression in birds and S-100 in mammals have been described in a wide variety of inflammatory, mesenchymal and visceral cell types, which may explain response to the wide range of disease conditions (So *et al*., 2009; Sekelova *et al*., 2017). Tissue and cell types expressing MRP-126 have not been characterized in reptiles; however, phylogenetic and syntenic analyses demonstrate that these proteins are highly conserved between species (Loes *et al*., 2018) and similar phenotypic expression is likely in sea turtles. Further studies characterizing MRP-126 expression in specific tissues and cells would provide further elucidation of the pathophysiology in sea turtles.

MRP-126 concentrations were negatively associated with PCV in both clinical studies. Regenerative (e.g. haemorrhage, hemolysis) and non-regenerative (e.g. anaemia of chronic disease, nutritional deficits from anorexia) anemias are commonly observed in stranded sea turtles (Casal and Orós, 2009; Deem *et al*., 2009; Stacy *et al*., 2018). Packed cell volume is associated with disease severity but can also demonstrate prolonged convalescence even when turtles are deemed clinically rehabilitated (Stacy *et al*., 2018). This pattern was evident within this study. For example, four of 12 (33%) clinically recovered juvenile loggerheads were considered anaemic when PCV percentages are compared with a regional reference interval of 17–39% (Kelly *et al*., 2015). MRP-126 concentrations categorized these turtles as healthy using the more conservative 2.1 ng/ml threshold. Additionally, four of 15 (27%) moribund loggerheads with a PCV that fell within the reference interval had elevated MRP-126 concentrations. This suggests that MRP-126 may represent a more reliable bioindicator for monitoring recovery during treatment than PCV alone.

MRP-126 was also negatively correlated with TS and BCS in the First and Second Clinical Studies, respectively. Both total solids and BCS are indicators of nutritional status (Labrada-Martagón *et al*., 2010; Melillo, 2013). This association likely reflects anorexia secondary to disease conditions. The lack of association with MRP-126 and BCI in the First Clinical Study may be secondary to the subjectivity of BCS compared to BCI, BCI differences between loggerheads and other turtle species (Nishizawa and Joseph, 2022) or variability in MRP-126 sensitivity by the SPARCL™ assay and ELISA. The lack of association between MRP-126 and TS in the Second Clinical Study may be affected by how the data were analysed. The last sample from turtles within the Serially Sampled Dataset was utilized to reduce variability of the data for statistical analysis. This may have skewed comparisons with MRP-126. The mean value for TS in the First Clinical Study was 3.3 g/dl, which is consistent with that described in debilitated loggerheads, compared to a mean of 4.0 g/dl in the Second Clinical Study, which is more aligned with concentrations described for healthy and recovered turtles (Deem *et al*., 2009; Molter *et al*., 2022).

Similar to trends observed with PCV, MRP-126 more correctly categorized health status in loggerhead turtles than TS when regional reference intervals of 4.4–5.7 d/dl were utilized (Molter *et al*., 2022). Two of five (40%) recovered and five of 15 (33%) moribund turtles were interpreted as hypoproteinemic. Of these turtles, five of five (100%) recovered and 14 of 15 (93%) moribund turtles were correctly classified using an MRP-126 concentration of 2.1 ng/ml as a threshold. The turtle (Cc1607) that was not correctly categorized as moribund presented with hook ingestion and demonstrated an MRP-126 concentration of 1.96 ng/ml similar to the 1.97 ng/ml threshold calculated by logistic regression suggesting this turtle was relatively clinically stable. MRP-126 also more correctly categorized health and disease than total white blood cell counts as three of five (60%) recovered turtles and four of 14 (29%) moribund turtles were assessed as leukopenic and glucose with hypoglycemia characterized in one of four (25%) moribund and three of 12 (25%) recovered turtles (Deem *et al*., 2009; Molter *et al*., 2022). MRP-126 shows promise as a single diagnostic biomarker for assessing the health of sea turtles and may provide greater utility for confirming recovery or convalescence during rehabilitation.

When CTNC was compared between groups, moribund turtles had significantly higher plasma concentrations than recovered turtles within the First Clinical Study and Second Clinical Study and moribund samples were significantly elevated compared to recovered samples in serially sampled turtles. Although both ROC and logistical regression were statistically significant models, CTNC was only marginally predictive of status. This dataset was relatively small due to limitations in reagent availability, and although CTNC may have potential as a clinical biomarker, a larger sample size is needed to further determine its value.

The premise behind evaluating CTNC concentrations was that it may offer a diagnostic assay to assess sea turtles presenting with etiologies associated with muscle damage but there were no clear associations with turtles presenting with trauma. The CTNC isoform examined in this study is specific to cardiac muscle and type 1 (oxidative) skeletal muscle and differs from skeletal troponin C (STNC), which is predominant in type 2 (glycolytic) skeletal muscle (Wang and Fuchs, 1994). The proportion of type 1 and 2 muscle fibres has not been examined in sea turtles but studies within freshwater red-eared sliders (*Trachemys scripta elegans*) demonstrate hind limb and neck muscles are predominantly type 2 (Callister *et al*., 2005) suggesting that STNC may represent a more suitable isoform for injuries to the neck and limb. The highest CTNC concentrations were observed in cold-stunned sea turtles with pneumonia suggesting secondary cardiac injury may be occurring, possibly from extension of inflammatory processes from the lungs to the heart or metabolic processes affecting heart function.

Cardiac troponin C was negatively correlated with BCI within the First Clinical Study. Cachexic states leading to decreased BCI are associated with skeletal muscle atrophy and adipose tissue loss (Patel and Patel, 2017). In humans, cachexia-induced muscle atrophy secondary to pathologic processes such as trauma, sepsis and neoplasia are associated with inflammatory signalling pathways that promote myofiber apoptosis and degradation (Ji *et al*., 2022) and increased muscle enzyme concentrations in plasma (Pérez-Peiró *et al*., 2022). Although reduced protein synthesis and inhibited amino acid uptake contribute as well, the observed correlation between increased CTNC and decreased BCI may be associated with similar catabolic processes. Although no correlation was present between CTNC concentrations and BCS in the Second Clinical Study, statistical comparisons between CTNC and BCI versus BCS may be constrained by similar species-specific analytic challenges as described for MRP-126 or secondary to the relatively small sample size.

Serum amyloid A concentrations in the First Clinical Study were 10× higher in moribund than recovered turtles but were also associated with a high IQR. The wide ranges observed between turtles within the same clinical grouping (i.e. recovered or moribund) are consistent with previous proteomic data that showed 100-fold differences between green turtles within the similar clinical classification (Marancik *et al*., 2021). The relatively high ranges in sea turtles may be a reflection of differences in disease severity in individual turtles although there were no clear associations between SAA concentrations and clinical parameters. There are numerous descriptions of SAA serum concentrations in domesticated mammals demonstrating much lower inter-individual variation during healthy and diseased states when animals are grouped together based on disease syndrome (Dąbrowski *et al*., 2013; Kaya *et al*., 2016; Wojtysiak *et al*., 2020). More in-depth examination of aetiologic-specific expression differences in sea turtles may provide specific conditions where SAA provides utility as a biomarker.

## Conclusions

SPARCL™ and ELISA methodologies provided targeted methodologies to quantify plasma concentrations of SAA, MRP-126 and CTNC in sea turtles. These assays represent novel strategies to improve our understanding of disease mechanisms as proteins have purported inflammatory, immunologic and physiologic roles. Analysing protein concentrations with variables such as health status, disease aetiology and life-stage class, and associations with clinical parameters, provide a clearer view of processes that influence inflammatory responses in sea turtles. MRP-126 shows promise as a complementary biomarker to evaluate sea turtle health, and future studies should be directed at developing reference intervals and examining expression and functional characteristics including its role in nesting physiology. Cardiac troponin C and SAA warrant further evaluation in larger sample sizes to examine variables affecting protein concentrations. Further progress in these areas continues to drive our understanding of sea turtle pathophysiology and validate the inclusion of low-cost biomarkers that provide insights into health status and enable more accurate diagnoses and monitoring within field and rehabilitation settings.

## Supplementary Material

Web_Material_coaf061

## Data Availability

The data underlying this article are available in the article and in its online Supplementary material.
